# A Nomogram for Predicting Brain Metastasis in IIIA-N2 Non-Small Cell Lung Cancer After Complete Resection: A Competing Risk Analysis

**DOI:** 10.3389/fonc.2021.781340

**Published:** 2021-12-13

**Authors:** Shuang Sun, Yu Men, Jingjing Kang, Xin Sun, Meng Yuan, Xu Yang, Yongxing Bao, Jianyang Wang, Lei Deng, Wenqing Wang, Yirui Zhai, Wenyang Liu, Tao Zhang, Xin Wang, Nan Bi, Jima Lv, Jun Liang, Qinfu Feng, Dongfu Chen, Zefen Xiao, Zongmei Zhou, Luhua Wang, Zhouguang Hui

**Affiliations:** ^1^ Department of Radiation Oncology, National Cancer Center/National Clinical Research Center for Cancer/Cancer Hospital, Chinese Academy of Medical Sciences and Peking Union Medical College, Beijing, China; ^2^ Department of Very Important Person (VIP) Medical Services, National Cancer Center/Cancer Hospital, Chinese Academy of Medical Sciences and Peking Union Medical College, Beijing, China; ^3^ Department of Radiation Oncology, National Cancer Center/Cancer Hospital and Shenzhen Hospital, Chinese Academy of Medical Sciences and Peking Union Medical College, Shenzhen, China

**Keywords:** nomogram, brain metastasis, non-small cell lung cancer, competing risk analysis, complete resection

## Abstract

**Background:**

Brain metastasis (BM) is one of the most common failure patterns of pIIIA-N2 non-small cell lung cancer (NSCLC) after complete resection. Prophylactic cranial irradiation (PCI) can improve intracranial control but not overall survival. Thus, it is particularly important to identify the risk factors that are associated with BM and subsequently provide instructions for selecting patients who will optimally benefit from PCI.

**Methods and Materials:**

Between 2011 and 2014, patients with pIIIA-N2 NSCLC who underwent complete resection in our institution were reviewed and enrolled in the study. Clinical characteristics, pathological parameters, treatment mode, BM time, and overall survival were analyzed. A nomogram was built based on the corresponding parameters by Fine and Gray’s competing risk analysis to predict the 1-, 3-, and 5-year probabilities of BM. Receiver operating characteristic curves and calibration curves were chosen for validation. A statistically significant difference was set as *P <*0.05.

**Results:**

A total of 517 patients were enrolled in our retrospective study. The median follow-up time for surviving patients was 53.2 months (range, 0.50–123.17 months). The median age was 57 (range, 25–80) years. Of the 517 patients, 122 (23.6%) had squamous cell carcinoma, 391 (75.6%) received adjuvant chemotherapy, and 144 (27.3%) received post-operative radiotherapy. The 1-, 3-, and 5-year survival rates were 94.0, 72.9, and 66.0%, respectively. The 1-, 3-, and 5-year BM rates were 5.4, 15.7, and 22.2%, respectively. According to the univariate analysis, female, non-smokers, patients with non-squamous cell carcinoma, bronchial invasion, perineural invasion, and patients who received adjuvant chemotherapy were more likely to develop BM. In a multivariate analysis, non-squamous cell carcinoma (subdistribution hazard ratios, SHR: 3.968; 95% confidence interval, CI: 1.743–9.040; *P* = 0.0010), bronchial invasion (SHR: 2.039, 95% CI: 1.325–3.139; *P* = 0.0012), perineural invasion (SHR: 2.514, 95% CI: 1.058–5.976; *P* = 0.0370), and adjuvant chemotherapy (SHR: 2.821, 95% CI: 1.424–5.589; *P* = 0.0030) were independent risk factors for BM. A nomogram model was established based on the final multivariable analysis result. The area under the curve was 0.767 (95% CI, 0.758–0.777).

**Conclusions:**

For patients with IIIA-N2 NSCLC after complete resection, a nomogram was established based on clinicopathological factors and treatment patterns for predicting the BM. Based on this nomogram, patients with a high risk of BM who may benefit from PCI can be screened.

## Introduction

Compared with early-stage non-small cell lung cancer (NSCLC), locally advanced NSCLC patients are more likely to develop brain metastasis (BM), nearly 30% of whom have BM within 2 years (1). Eight randomized controlled trials of prophylactic cranial irradiation (PCI) in NSCLC suggested that PCI was associated with a decrease in the risk of BM but that its performance was still unsatisfactory in improving survival (2–9). However, a randomized phase III study (4) with fully resected stage IIIA-N2 NSCLC patients showed that PCI can lengthen the disease-free survival. It demonstrated a positive effect of screening patients with BM high-risk metastasis, which merits further study. This study is aimed to screen pIIIA-N2 NSCLC individuals with high-risk BM in order to facilitate further randomized PCI studies in the future.

In previous studies, BM was often regarded as the primary end event, but the impact of death on BM was ignored. A competing risk models is a combination of two or more distributions that represent failure modes that are “competing” to be the end event of the system that is being modeled. This model considers the effects of the other competed risks, so it can estimate the probability of the primary endpoint event concurrence more accurately. The nomogram model can visualize the regression model and intuitively expresses the influence of those related factors on the event. Because of its simplicity, visibility, and high efficiency, it has been used in medical research.

## Materials and Methods

### Patient Selection and Data Collection

In this validation study, the eligibility criteria were as follows: (i) patients who underwent surgery in our institution between January 2011 and December 2014, (ii) patients who received complete resection and systemic lymph node dissection, (iii) patients who were pathologically diagnosed with stage IIIA-N2 NSCLC, according to the seventh edition of the American Joint Committee on Cancer Staging System, and (iv) patients for whom complete medical records were available. The exclusion criteria were as follows: (i) patients who were treated with prophylactic cranial irradiation after surgery, (ii) patients with a previous history of cancer in the past or combined with a second tumor, and (iii) patients who received anti-tumor therapy before surgery. The study variables included age, sex, smoking status, tumor location, surgery type, pathological features, number of positive lymph nodes, and postoperative treatment.

### Treatment

#### Surgery

Complete resection included lobectomy, bilobectomy, or pneumonectomy with complete exploration and dissection of the mediastinal lymph nodes at levels 4, 7, and 10 for right lung cancer and at levels 4 (if accessible), 5, 6, 7, and 10 for left lung cancer.

#### Postoperative Radiotherapy

The radiotherapy techniques included three-dimensional conformal radiotherapy or intensity-modulated conformal radiotherapy. The clinical target volume (CTV) included the ipsilateral hilum, subcarinal region, and ipsilateral mediastinum. The stumps of the central lesions were also included in the CTV. Postoperative radiotherapy was administered with 6-MV X-rays at 2 Gy per fraction for up to 50 Gy over 5 weeks.

#### Adjuvant Chemotherapy

Adjuvant chemotherapy was administered with four cycles of a platinum-based doublet regimen.

### Follow-Up

The patients were followed up every 3 months for the first 2 years, every 6 months for 2–5 years, and once per year after 5 years. During follow-up, all patients were evaluated with blood examination, chest CT, abdominal CT or B-ultrasound, and other necessary examinations based on their symptoms. The patients underwent enhanced MRI examination of the brain every 6 months for 5 years and when they had suspicious symptoms. Enhanced CT was required for MR-contraindicated patients. Disease progression was confirmed by clinical assessments, radiologic examination, or pathology reports. Both the initial and the subsequent sites of recurrence were documented. All patients were followed for at least two years, except those with intracranial progression or those who died.

### Statistical Analyses

The primary index was BM, which was defined as an intracranial recurrence that was diagnosed by MRI or CT throughout the course of the disease, and the time to BM was defined as the time interval between the date of surgical resection and BM or the last follow-up. The secondary outcome measurement was overall survival, which was measured from the date of surgery to death.

“BM” was regarded as the ending event and “death” that was caused by cancer, loco-regional progression, comorbidity”, or any other reason except that BM was regarded as the competing event. The censored events were those who got lost to follow-up and with no occurrence of either the ending event or the competing event after the end of the trial. The cumulative incidence of BM was estimated by the cumulative incidence function. Risk factors for BM were analyzed by the Fine and Gray proportional sub-distribution hazards regression. A prediction model was established based on the independent risk factors, and its prediction ability and clinical practicability were evaluated. The discrimination ability of the multivariate Cox model for predicting the 3- and 5-year probabilities of developing BM was assessed by a concordance measure that is analogous to the area under the receiver operating characteristic (ROC) curve. We plotted calibration curves to evaluate the prediction efficiency of this model. Statistical analyses were performed using IBM SPSS Statistics, version 25.0, and R version 4.0.5 (http://www.r-project.org/). All statistical tests were bilateral tests, and differences between the data were expressed in terms of the *P*-value, and *P <*0.05 was considered statistically significant.

## Results

### Patient Characteristics and Recurrence Pattern

A total of 517 patients were enrolled in our study. The median follow-up time for surviving patients was 53.17 months (range, 5.23–123.17 months). The median age was 57 years old (range, 25–80 years). Of the 517 patients, 122 (23.6%) had squamous cell carcinoma (SCC), 391 (75.6%) received adjuvant chemotherapy, and 144 (27.3%) received postoperative radiotherapy. The details of patient characteristics and treatment patterns are presented in **Table 1**.

**Table 1:** Patients characteristics and treatment pattern.

Factors	No. Of patients	%
**Age, years**		
<60	313	60.5
≥60	204	39.5
**Sex**		
Male	315	60.9
Female	202	39.1
**Smoking status**		
Smoker	247	47.8
Non-Smoker	270	52.2
**Histology**		
Non-SCC	395	76.4
SCC	122	23.6
**Necrosis**		
Yes	90	17.4
Unknown	427	82.6
**Pleural invasion**		
Yes	351	67.9
No	166	32.1
**Bronchial invasion**		
Yes	269	52.0
No	248	48.0
**Vascular invasion**		
Yes	62	12.0
No	455	88.0
**Intravascular cancer embolus**		
Yes	107	20.7
No	410	79.3
**Perineural invasion**		
Yes	26	5.0
No	491	95.0
**pT**		
T1	77	14.9
T2-3	440	85.1
**NMLN**		
>4	280	54.2
≤4	237	45.8
**LNR**		
≥30%	192	37.1
<30%	325	62.9
**Adjuvant chemotherapy**		
Yes	391	75.6
No	126	24.4
**PORT**		
Yes	141	27.3
No	376	72.7

BM, Brain metastasis; SCC, Squamous cell carcinoma; NMLN, Number of lymph node metastases; LNR, lymph node ratio; PORT, Postoperative radiotherapy.

A total of 365 patients were still alive until the last follow-up time. The 1-, 3-, and 5-year survival rates were 94.0, 72.9, and 66.0%, respectively. Of the 517 patients, 289 (55.9%) patients had disease progression, 128 (24.8%) patients had loco-regional recurrence, and 240 (46.4%) patients had distant metastasis. A total of 95 patients (18.4%) had BM. Metastasis to the brain as the first relapse site occurred in 64 patients (12.4%). The 1-, 3-, and 5-year BM rates were 5.4, 15.7, and 22.2%, respectively.

### Risk Factors for BM

According to the univariate analysis that was based on the competing risk model, females, non-smokers, patients with non-squamous cell carcinoma, bronchial invasion, perineural invasion, and patients who received adjuvant chemotherapy were more likely to develop BM (**Table 2**). According to a multivariate analysis, non-squamous cell carcinoma (subdistribution hazard ratios, SHR: 3.968, 95% confidence interval, CI: 1.743–9.040; *P* = 0.0010), bronchial invasion (SHR: 2.039, 95% CI: 1.3253.139; *P* = 0.0012), perineural invasion (SHR: 2.514, 95% CI: 1.058–5.976; *P* = 0.0370), and adjuvant chemotherapy (SHR: 2.821, 95% CI: 1.424–5.589; *P* = 0.0030) were independent risk factors for BM (**Table 3**).

**Table 2:** Univariate competing risk model analysis of brain metastasis in patients with resected IIIA-N2 NSCLC.

Factors	BM				Death			
	1y(%)	3y(%)	5y(%)	P-value	1y(%)	3y(%)	5y(%)	P-value
**Age, years**								
<60	5.4	14.6	20.1	0.407	3.5	18.7	23.6	0.002
≥60	4.9	13.7	16.9		9.3	27.3	35.1	
**Sex**								
Male	4.4	11.5	13.9	0.001	8.2	27.5	33.7	<0.001
Female	6.4	18.4	26.7		2.0	13.7	19.2	
**Smoking status**								
Smoker	4.1	10.4	11.4	<0.001	8.5	28.6	34.3	<0.001
Non-Smoker	6.4	18.4	27.3		2.8	14.9	21.1	
**Histology**								
Non-SCC	6.1	17.4	23.0	<0.001	5.1	17.4	23.7	<0.001
SCC	2.4	4.1	6.1		8.2	36.8	41.7	
**Necrosis**								
Yes	6.7	13.5	18.6	0.566	10.0	35.5	41.9	0.001
Unknown	4.9	14.3	19.1		4.9	19.2	25.2	
**Pleural invasion**								
Yes	6.3	14.9	21.6	0.078	6.0	22.8	30.6	0.301
No	3.0	12.9	13.6		5.4	20.6	23.1	
**Bronchial invasion**								
Yes	7.4	16.6	21.9	0.034	6.4	25.2	30.2	0.174
No	2.8	11.6	15.5		5.2	18.6	25.7	
**Vascular invasion**								
Yes	4.8	9.7	9.7	0.074	3.2	25.0	27.2	0.363
No	5.2	14.8	20.0		6.1	21.7	28.1	
**Intravascular cancer embolus**								
Yes	7.5	19.6	23.1	0.278	7.4	30.8	32.6	0.144
No	4.6	12.9	17.8		5.4	19.9	25.8	
**Perineural invasion**								
Yes	15.4	23.1	28.8	0.044	3.8	19.7	28.3	0.846
No	4.7	13.8	18.3		5.9	22.2	28.1	
**pT**								
T1	<0.001	10.4	12.1	0.218	6.5	17.9	19.7	0.169
T2-3	6.1	14.9	20.2		5.7	22.8	29.6	
**NMLN**								
>4	7.1	17.5	22.5	0.069	7.1	24.1	30.3	0.116
≤4	3.0	10.3	14.6		4.2	19.7	25.3	
**LNR**								
≥30%	4.6	13.1	17.1	0.332	4.6	19.6	24.7	0.029
<30%	6.3	16.1	22.1		7.8	26.4	33.9	
**Adjuvant chemotherapy**								
Yes	6.4	16.9	22.0	<0.001	2.8	17.3	21.4	<0.001
No	1.6	5.8	9.0		15.1	37.3	50.3	
**PORT**								
Yes	5.0	18.2	24.9	0.162	<0.001	11.2	17.1	<0.001
No	5.3	12.7	16.5		7.9	26.2	32.2	

BM, Brain metastasis; SCC, Squamous cell carcinoma; NMLN, Number of lymph node metastases; LNR, lymph node ratio; PORT, Postoperative radiotherapy.

**Table 3:** Regression coefficients in Fine and Gray model for BM.

Factor	SHR	95% CI	P value
**Sex**	1.13	0.655-1.96	0.6600
**Smoking status**	1.84	0.967-3.50	0.0630
**Histology**	3.968	1.743-9.04	0.0010
**Bronchial invasion**	2.039	1.325-3.139	0.0012
**Perineural invasion**	2.514	1.058-5.976	0.0370
**Adjuvant chemotherapy**	2.821	1.424-5.589	0.0030

### Prediction Model and Nomogram for BM

**Figure 1:**
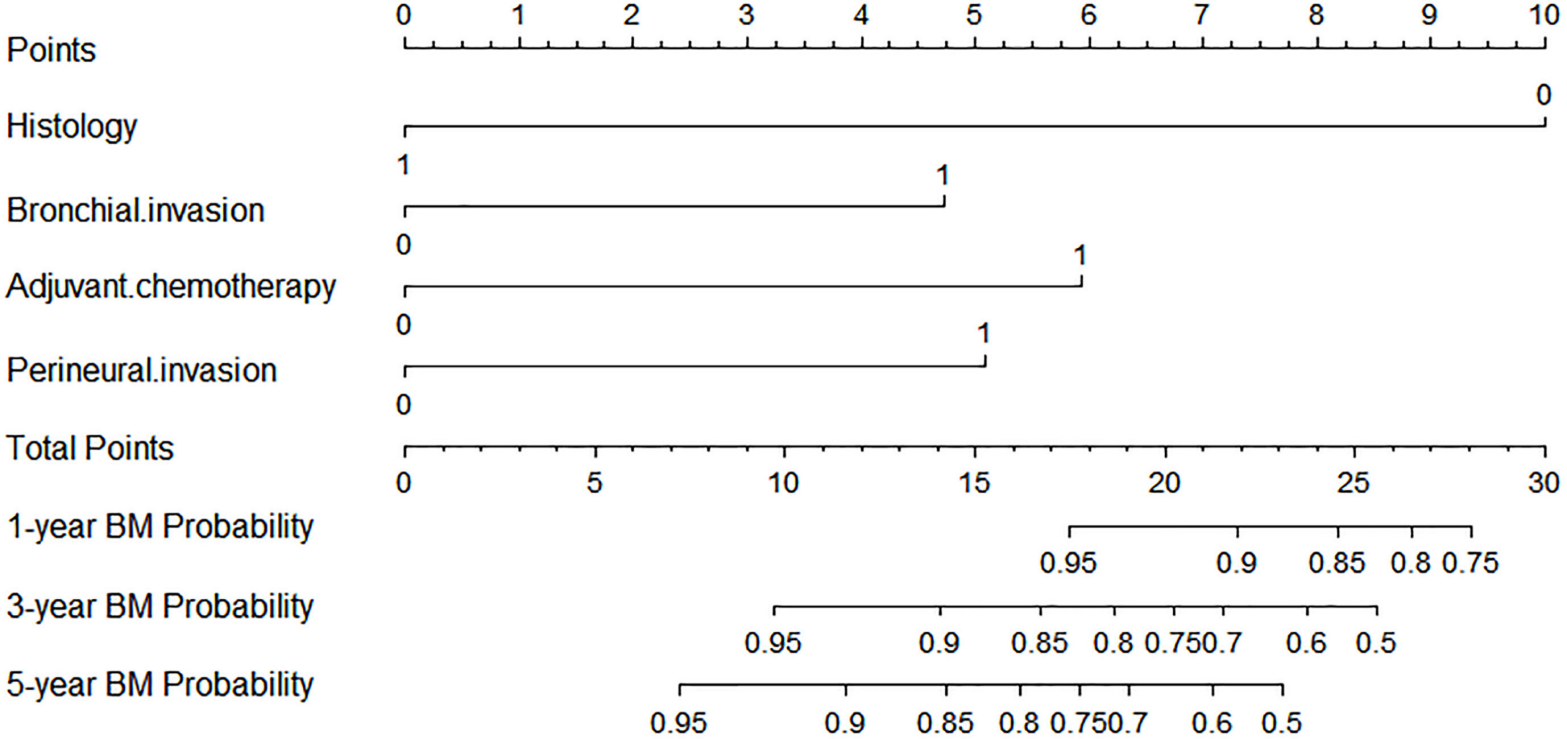
Competing risk nomogram for predicting the 1-, 3-, and 5-year probabilities of brain metastasis in patients with resected IIIA-N2 non-small cell lung cancer.

**Figure 2:**
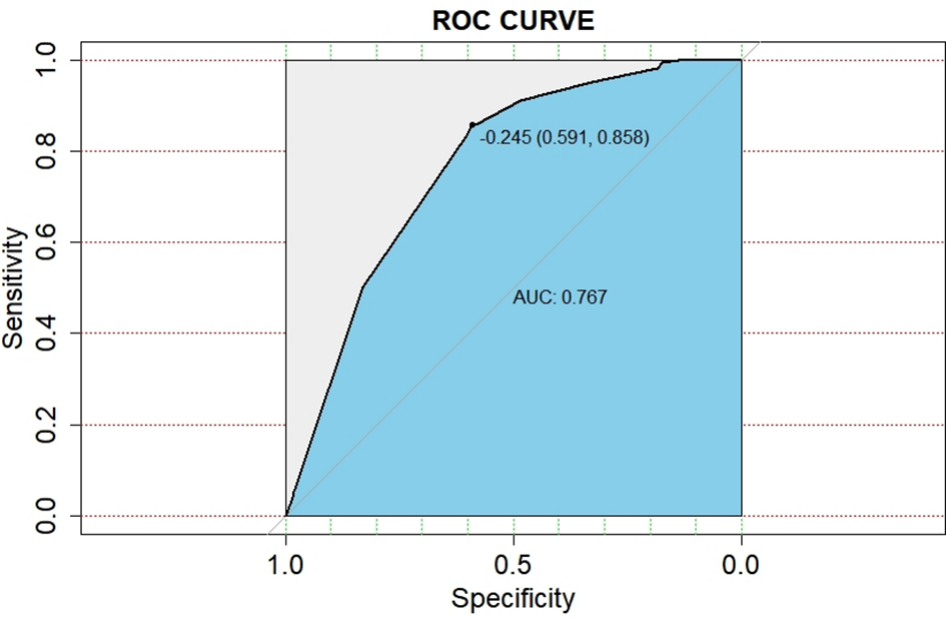
Receiver operating characteristic curve for predicting the probabilities of brain metastasis in patients with resected IIIA-N2 non-small cell lung cancer.

**Figure 3:**
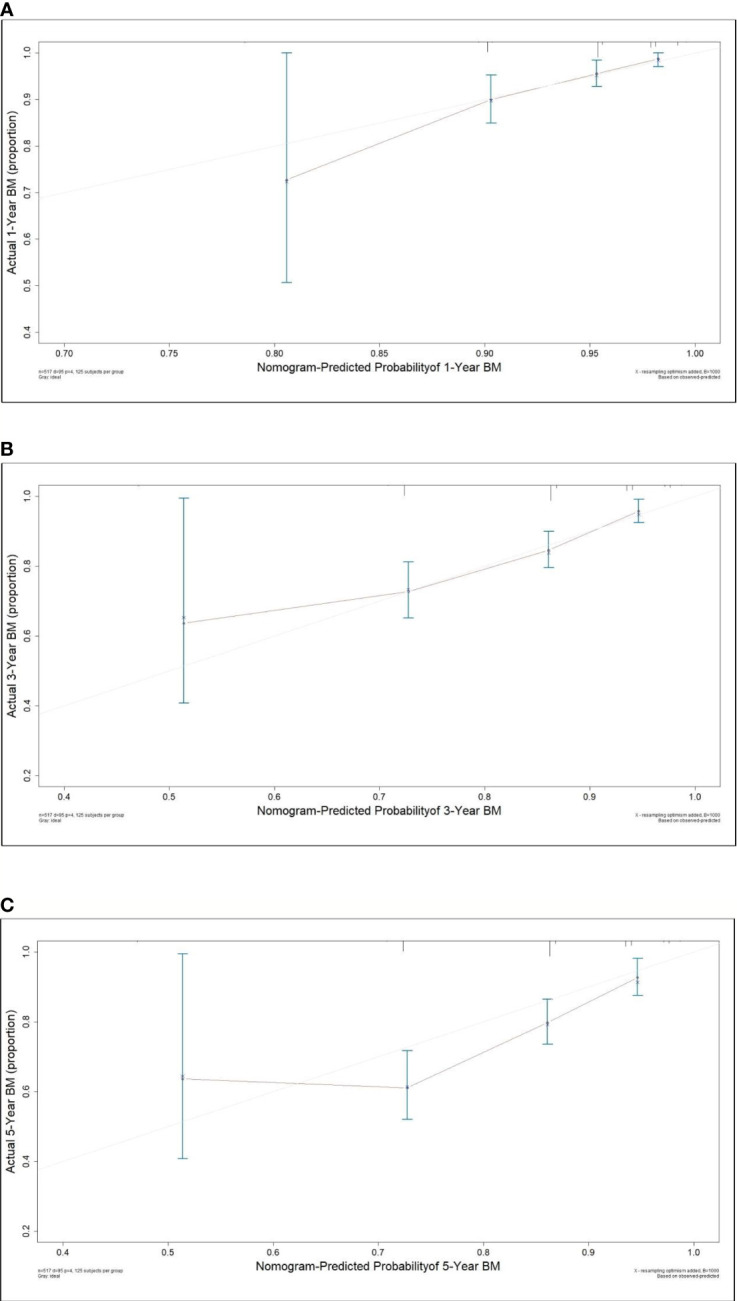
Calibration curve for predicting the 1- **(A)**, 3- **(B)** and 5-year **(C)** probabilities of brain metastasis in patients with resected IIIA-N2 non-small cell lung cancer.

A nomogram model was established based on the multivariable analysis results (**Figure 1**). The ROC curve of this prediction model is shown in **Figure 2**, and the area under the curve (AUC) was 0.767 (95% CI, 0.758–0.777). Calibration curves that were obtained based on bootstrap resampling validation are plotted in **Figure 3**.

## Discussion

To the best of our knowledge, this study was the first and largest population-based study to develop a nomogram model for predicting the occurrence of BM in pIIIA-N2 NSCLC by competing risk analysis, in which the bias that is caused by death is considered. The standard Cox regression does not account for the competing risk of death; thus, it underestimates the risk of BM of patients who arrive at the competing event first. The developed nomogram for predicting the 1-, 3-, and 5-year probabilities of BM in IIIA-N2 NSCLC patients is based on independent factors, including histology, bronchial invasion, perineural invasion, and adjuvant chemotherapy. The predictive ability of this competing risk model was demonstrated for the entire risk range and prediction time range, according to the AUC (0.767; 95% CI: 0.758–0.777). The calibration plot showed the discrimination performance of the nomogram by internal bootstrap resampling validation.

Patients with IIIA-N2 have a high risk of death. Complete resection followed by adjuvant chemotherapy or sequential chemoradiotherapy is recommended for those patients according to the newly published National Comprehensive Cancer Network guidelines (10). There is a reason to believe that pIIIA-N2 patients are the cohort who received the most radical treatment for extracranial lesions among locally advanced NSCLC patients. Reducing the risk of intracranial death has become the focus of current research. Therefore, accurately identifying and selecting the BM high-risk population who may benefit from PCI is very important. However, the previously established nomogram models for predicting BM in NSCLC all focus on patients with stage I–III disease. Won et al. developed a prediction model that considers five clinical factors, including histology, smoking status, pT stage, and the interaction between adenocarcinoma and pN stage, and the C-statistics for 2 and 5 years were 69.3% (95% CI, 62.6–76.0%) and 69.8% (95% CI, 63.6–75.9%), respectively (11). According to neuron-specific enolase level, histological type, number of metastatic lymph nodes, and tumor grade, Zhang et al. established a nomogram for calculating the BM risk at 3 and 5 years (C-index 0.74; 95% CI: 0.67–0.82) (12). Wang et al. analyzed the patients with stage III NSCLC and reported the following mathematical model of BM risk: Logit (*P*) = 8.215 − 0.903 × (number of metastases lymph node) − 0.872 × (surgical resection evaluation) − 0.714 × (histology) − 1.893 × (regional LN metastases) − 0.948 × (TNM-stage) − 1.034 × (adjuvant chemotherapy). ROC testing demonstrated that, when *P* = 0.44, both the sensitivity and specificity reached their maximum values (sensitivity = 80% and specificity = 77%) (13). Compared with previous studies, our study focused mainly on the most heterogeneous stage IIIA-N2 patients. Meanwhile, we used the competing risk model to accurately assess the incidence of BM. In addition to clinical factors, postoperative pathological tumor infiltration and postoperative treatments were also taken into account. Our study is consistent with these three studies in identifying histological types as important prognostic factors. We also identified three new prognostic factors: bronchial invasion, perineural invasion, and adjuvant chemotherapy. The predictive performance of this competing risk model was demonstrated for the entire risk range and prediction time, according to AUC (0.767, 95% CI: 0.758–0.777).

Histology was a strong predictor of BM in our study, and patients with non-SCC were more likely to develop BM. This was also reported in previous studies (5, 11–16). In recent years, some studies have found that the existence of a micropapillary pattern in adenocarcinoma, which accounts for the most proportion of non-SCCs, increases the ability of cancer cells to invade blood vessels. The high proliferation rate of tumors leads to hypoxic necrosis of cancer cells, which causes the upregulation of angiogenesis genes. This may increase the risk of BM and shorten the time to BM (17, 18). In addition, non-SCC exhibits mainly invasive growth, and the likelihood of hematogenous metastasis is relatively high, which may also be a reason for BM.

A study from the Radiation Therapy Oncology Group showed that that adjuvant chemotherapy increased the BM rate of patients with locally advanced non-small cell lung cancer (19). Liang et al. retrospectively analyzed 193 patients with completely resected stage IIIA NSCLC and found that the 3-year BM rates were 47.3% for patients with adjuvant chemotherapy and 30.5% for those without adjuvant chemotherapy (20). Wang et al. showed that there was a significant difference in BM frequency according to absence *versus* administration of adjuvant chemotherapy (*P* = 0.032) (13). After considering the competing risk of death, our study also showed that adjuvant chemotherapy can increase the risk of BM. The BM rates of 1, 3, and 5 years were 6.4 *vs*. 1.6%, 16.9 *vs*. 5.8%, and 22.0 *vs*. 9.0%, respectively (*P* = 0.029). Patients who receive chemotherapy may have a higher likelihood of BM occurrence observation due to the longer survival time they may obtain.

Our study also found that bronchial invasion and perineural invasion are also the high-risk factors for BM. However, no relevant research has been reported. The majority of tumors with bronchial invasion are adenocarcinoma, which may be related to BM.

This study had various limitations. First, this study was a single-center study. In contrast, a multicenter study may be more representative of the general population and provide more convincing results. Second, the epidermal growth factor receptor and programmed cell death 1 ligand 1 status is not included in the analysis. The main reason for the lack of gene status data in this study is that the detection of driving gene status was not normalized since 2011 so that the amount of data was limited in this study. Third, the number of events was quite low. This study used bootstrap resampling method for internal validations; however, external validation may increase the robustness of the model. Although it is a common method, it cannot fully guarantee the applicability to external data.

## Conclusions

For patients with IIIA-N2 NSCLC after complete resection, a nomogram was established based on the clinicopathological factors and treatment pattern for predicting the BM. Based on this nomogram, patients with a high risk of BM who may benefit from PCI can be identified.

## Data Availability Statement

The original contributions presented in the study are included in the article/supplementary material. Further inquiries can be directed to the corresponding author.

## Author Contributions

SS, YM, JK, XS, MY, XY, and YB collected raw data. SS checked the data, performed statistical analysis, and drafted the manuscript. ZH revised the manuscript. All authors contributed to the article and approved the submitted version.

## Funding

This work was supported by the National Key Research and Development program (2017YFC1311000 and 1311002), Clinical Application Project of Beijing Municipal Commission of Science and Technology (Z171100001017114), and Beijing Hope Run Special Fund for Medical Sciences (LC2016L03).

## Conflict of Interest

The authors declare that the research was conducted in the absence of any commercial or financial relationships that could be construed as a potential conflict of interest.

## Publisher’s Note

All claims expressed in this article are solely those of the authors and do not necessarily represent those of their affiliated organizations, or those of the publisher, the editors and the reviewers. Any product that may be evaluated in this article, or claim that may be made by its manufacturer, is not guaranteed or endorsed by the publisher.
